# Imaging furcation defects with low-dose cone beam computed tomography

**DOI:** 10.1038/s41598-022-10781-0

**Published:** 2022-04-26

**Authors:** Maurice Ruetters, Holger Gehrig, Ti-Sun Kim, Valentin Bartha, Thomas Bruckner, Franz Sebastian Schwindling, Anna Felten, Christopher Lux, Sinan Sen

**Affiliations:** 1 Section of Periodontology, Department of Operative Dentistry, University Hospital Heidelberg, Im Neuenheimer Feld 400, 69120 Heidelberg, Germany; 2 Institute of Medical Biometry, University Hospital Heidelberg, Im Neuenheimer Feld 130.3, 69120 Heidelberg, Germany; 3 Department of Prosthetic Dentistry, University Hospital Heidelberg, Im Neuenheimer Feld 400, 69120 Heidelberg, Germany; 4 Department of Orthodontics, University Hospital Heidelberg, Im Neuenheimer Feld 400, 69120 Heidelberg, Germany; 5 Department of Orthodontics, University Hospital Schleswig Holstein, Arnold-Heller-Straße 3, 24105 Kiel, Germany

**Keywords:** Oral diseases, Periodontitis

## Abstract

Different cone beam computed tomography (CBCT) protocols have shown promising results for imaging furcation defects. This study evaluates the suitability of low-dose (LD)-CBCT for this purpose. Fifty-nine furcation defects of nine upper and 16 lower molars in six human cadavers were measured by a high-dose (HD)-CBCT protocol, a LD-CBCT protocol, and a surgical protocol. HD-CBCT and LD-CBCT measurements were made twice by two investigators and were compared with the intrasurgical measurements, which served as the reference. Furcation defect volumes generated from HD-CBCT and LD-CBCT imaging were segmented by one rater. Cohen’s kappa and intraclass correlation coefficient (ICC) values were calculated to determine intra- and interrater reliability. The level of significance was set at α = 0.05. In total, 59 furcation defects of nine upper and 16 lower human molars were assessed. Comparing CBCT furcation defect measurements with surgical measurements revealed a Cohen’s kappa of 0.5975 (HD-and LD-CBCT), indicating moderate agreement. All furcation defects identified by HD-CBCT were also detected by LD-CBCT by both raters, resulting in a Cohen’s kappa of 1. For interrater agreement, linear furcation defect measurements showed an ICC of 0.992 for HD-CBCT and 0.987 for LD-CBCT. The intrarater agreement was 0.994(r1)/0.992(r2) for HD-CBCT and 0.987(r1)/0.991(r2) for LD-CBCT. The intermodality agreement was 0.988(r1)/0.991(r2). Paired t-test showed no significant differences between HD-CBCT and LD-CBCT measurements. LD-CBCT is a precise and reliable method for detecting and measuring furcation defects in mandibular and maxillary molars in this experimental setting. It has the potential to improve treatment planning and treatment monitoring with a far lower radiation dose than conventional HD-CBCT.

## Introduction

Knowing the specific anatomy of a furcation defect helps optimize prognosis and periodontal treatment planning^1^. The Nabers probe has been used to detect furcation defects, but the complex anatomy of these defects can interfere with the stiffness and shape of the probe, which limits the ability of the probe to define the anatomy^2^. To overcome these limitations, further imaging diagnostics are performed, but these methods are not three-dimensional. Although two-dimensional imaging can determine whether a furcation defect is continuous or not in mandibular molars, this is almost impossible in maxillary molars. Two-dimensional imaging of the complex anatomy and superimposition of the roots in maxillary molars agrees only slightly with that of three-dimensional imaging^3^.

Three-dimensional imaging using cone beam computed tomography (CBCT) can reliably depict this complex anatomy. Walter et al. investigated the agreement of CBCT with presurgical and intrasurgical measurements and showed that CBCT can clearly visualize furcation defects^4^. Another study confirmed that obtaining a CBCT when planning periodontal surgery can make treatment more patient-specific, which can reduce costs^5^. However, CBCT exposes the patient to higher radiation doses than conventional two-dimensional procedures (intraoral radiographs and panoramicradiographs) do^6,7^.

Newer CBCT devices can lower the radiation dose, for example by using pulsed radiation, shortening the scan time, and reducing the radiation source orbit to 180°^6^. Manufacturer-dependent, these low-dose (LD)-CBCT protocols can reduce the dose-area product (DAP) by up to 90% compared with HD-CBCT protocols from the same device^8,9^. These DAPs are similar to those of a panoramic radiograph and, in some cases, are even lower than the radiation dose of a full-mouth status^10^. However, lowering the radiation dose can reduce image quality. Studies have shown that LD-CBCT can visualize delicate structures such as the buccal and oral bone lamellae^11^. In the present study, we examined whether LD-CBCT can detect and measure furcation defects. We hypothesized that LD-CBCT would detect furcation defects with the same image quality as HD-CBCT.

## Materials and methods

This ex vivo study examined nine maxillary and 16 mandibular molars in six human cadaveric heads. The bodies were donated to the Institute of Anatomy and Cell Biology at Heidelberg University Hospital. The cadavers were preserved in 99% ethanol, glycerin, and 37% formalin.

### Cone beam computed tomography

At the time of radiographic investigation, the human heads included mandibles that were fully covered by soft tissue and adjacent cheek muscles. The tongue, neck muscles, skull base, and cervical vertebrae were also still present. The teeth were investigated radiographically by LD-CBCT and HD-CBCT using the same CBCT device (Orthophos 3D SL ®, DentsplySirona, Bensheim, Germany).

For HD-CBCT, volumetric acquisition was performed under the following conditions: radiation exposure time, 14.2 s; 6 mA; 85 kV; field of view, 8 × 8 cm^2^; isotropic voxel size, 0.16 mm; DAP, 943 mGy cm^2^. For LD-CBCT, volumetric acquisition was performed under the following conditions: radiation time, 2.1 s; 10 mA; 85 kV; field of view, 8 × 8 cm^2^; isotropic voxel size, 0.16 mm; DAP, 67 mGy cm^2^.

During volumetric recordings, the heads were fixed with their throat in a tube and orientated according to the manufacturers’ orientation lines. In total, 12 scans were taken for analysis.

### Surgical examination

After the CBCT images were taken, any soft tissue covering the furcations was removed and any granulation tissue was cleared from the defects using hand instruments. Then, furcation was measured using a Nabers probe with a 3 mm scale (PQ2N; HU-Friedy) as previously described^12^.

### Image review

**Figure 1:**
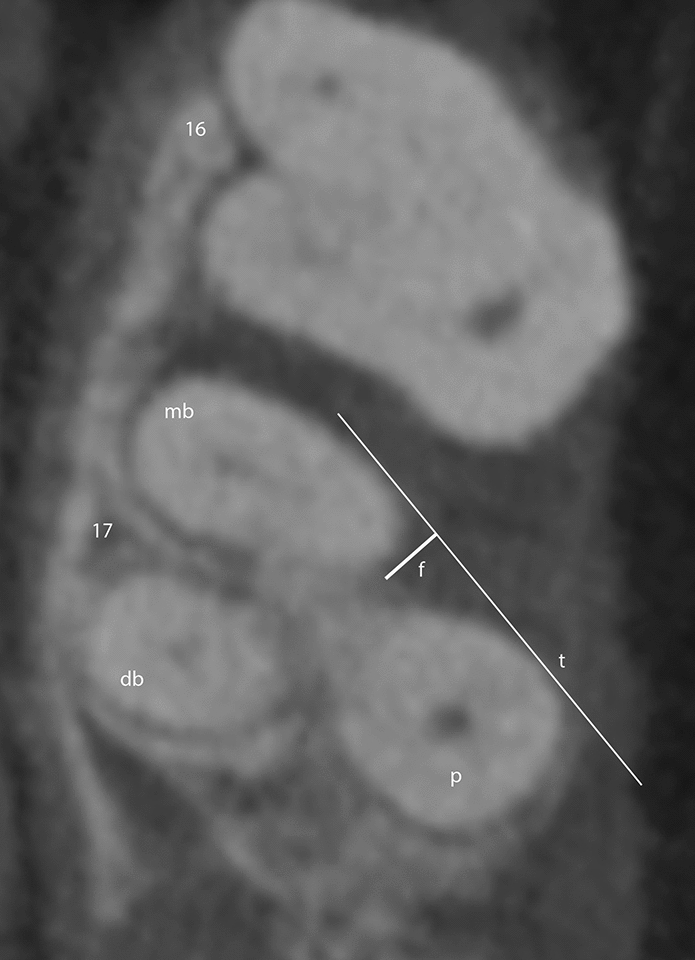
Measurement at the mesiopalatal furcation entrance of tooth 17 in the axial plane. *mb* mesiobuccal, *db* distobuccal, *p* palatinal, *t* tangent line along the outermost superficial points of the two limiting roots, *f* distance from the tangent line to the furthest point of the furcation defect.

**Figure 2:**
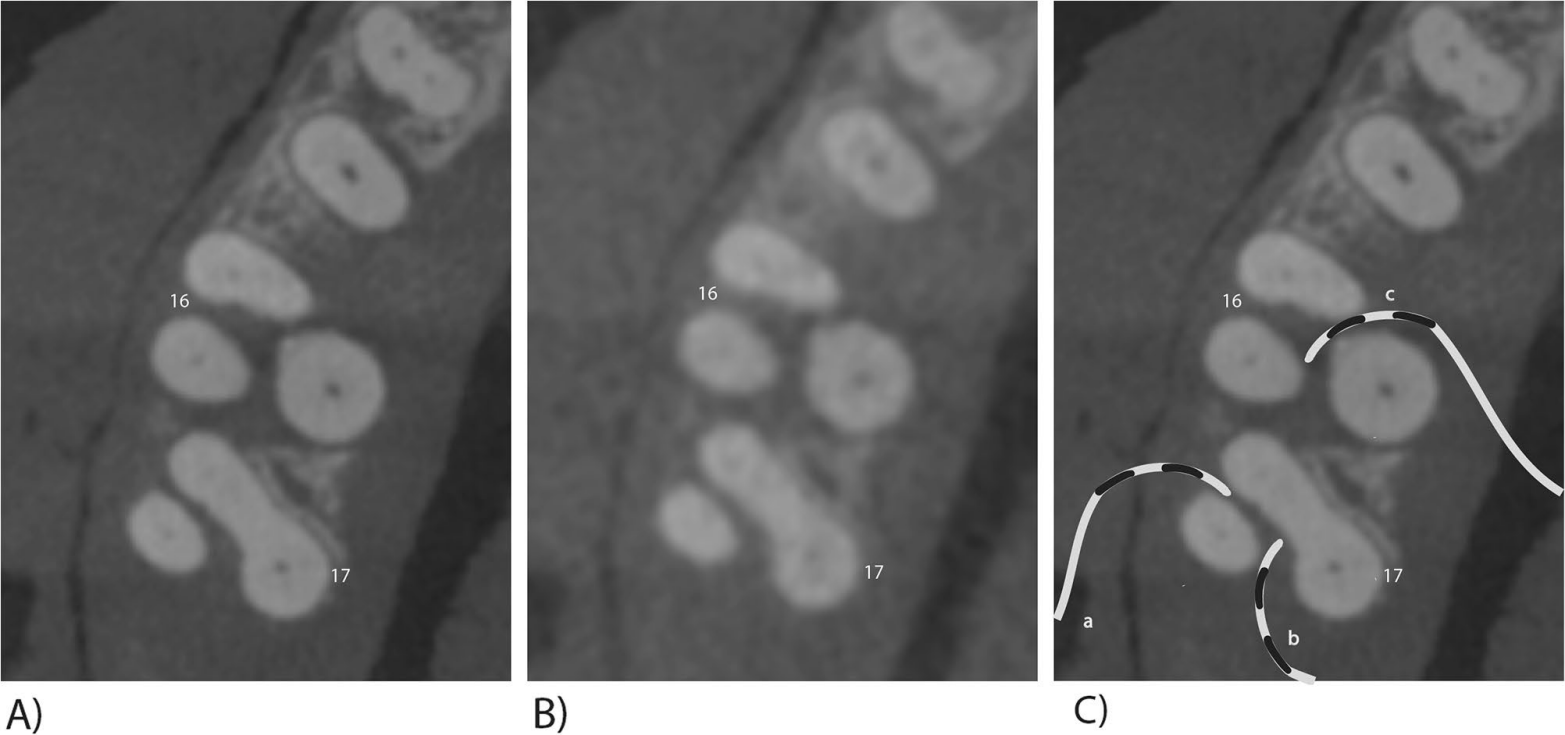
Different CBCT modes of teeth 16 and 17 with furcation defects. (**A**) HD-CBCT (**B**) LD-CBCT (**C**)**. a**–**c**: different problematic intrasurgical probing angles leading to underestimation of the furcation defect.

CBCT data was exported in DICOM format for analysis using the application software OSIRIX pro (aycanOsiriX 2.06.000). Windowing and leveling were allowed. Images were evaluated on the same monitor (iMac, 27ʺ, Apple, California, USA) in the same dark room. Images were reviewed independently by two dentists (M.R., H.G.) with eight (M.R.) and 15 years (H.G.) of experience in CBCT diagnostics. The reviewers were blinded to the surgical furcation measurements in multiplanar reformations. Each furcation defect was assessed in all three planes and the reviewer also determined whether the defect was a continuous third-degree furcation defect or a non-continuous first- or second-degree furcation defect. If the defect was non-continuous, a tangent line was also drawn in the horizontal plane along the outermost superficial points of the two limiting roots in a defined image, and the distance (f) from the tangent line (t) to the furthest point of the furcation defect was measured orthogonally (Fig. 1). The measured line was defined on each image to ensure that both examiners measured in the same plane during both HD-CBCT and LD-CBCT. The measurements were used to define the clinical grade (grade 0: no furcation defect, grade I: defect less than 3 mm, grade II: defect more than 3 mm, grade III: defect continuous) (Fig. 2). Each measurement was taken twice for each protocol with an interval of two weeks between the measurements to exclude a memory effect. A total of 60 furcation entrances were examined twice each by HD-CBCT and LD-CBCT by each examiner, giving a total number of 240 observations per examiner and 480 observations in total.

For calibration, 30 similar measurements were made from different CBCTs to the ones used in this study. Any deviations were discussed until agreement was achieved.

Furcation defects were also segmented manually and these segmentations were used to calculate the furcation volume in defined contiguous layers with HD-CBCT and LD-CBCT and to assess the volume reproducibility. The segmentation was done by one experienced investigator (M.R.) using the hardware and software described above. After segmentation, STL datasets were exported from Osirix and imported into a matching software (Geomagic Design X, (3D Systems; Rock Hill, South Carolina, U.S.A.) for better visualization.

### Statistical analysis

The sample number was restricted to the teeth available, so the sample size was not calculated. Interrater and intrarater agreement was measured using the intraclass correlation coefficient (ICC) with 95% confidence interval. Measurements were presented descriptively as medians with neighboring quartiles (Q1 and Q3). Cohen’s kappa was used to measure the correlation of CBCT-measurements with clinical examinations. The paired t-test was used to determine whether differences were significant. The significance level was set at α = 0.05.

For statistical analysis, SAS software version 9.4win was used.

### Ethical approval

All procedures performed in studies involving human participants were in accordance with the ethical standards of the institutional and national research committee and with the 1964 Helsinki declaration and its later amendments or comparable ethical standards. The study was approved by the ethical review board of the University of Heidelberg, Germany (S-017/2020).

### Informed consent

All human donors have already given their consent beforehand for donation of their body to the Department of Anatomy and Cell Biology of the University of Heidelberg for research. The ethical committee of the University of Heidelberg assured that the informed consent, they already fave for scientific reasons, is absolutely fine and covers this study.

## Results

In total, 59 furcation entrances and possible furcation defects of nine upper and 16 lower human molars were assessed radiographically using HD-CBCT and LD-CBCT (Table 1). Intrasurgical measurements revealed 33 sites at 16 molars with furcation defects. Six sites with grade III furcation defects were identified at three lower molars and 21 sites with grade I and II furcation defects were identified at 13 molars (seven upper and six lower molars).

**Table 1:** (a) Means and medians of linear furcation measurements (f); (b) ICC-values of linear furcation measurements (f).

Protocol	Mean	Std Dev	Min	Median	Max
**(a)**					
*HD-CBCT R1 1*	1.78	0.94	0.61	1.44	4.26
*LD-CBCT R1 1*	1.82	0.95	0.63	1.48	4.36
*HD-CBCT R1 2*	1.80	0.95	0.76	1.49	4.18
*LD-CBCT R1 2*	1.79	0.91	0.76	1.47	4.11
*HD-CBCT R2 1*	1.79	0.93	0.78	1.49	4.31
*LD-CBCT R2 1*	1.80	0.97	0.80	1.44	4.31
*HD-CBCT R2 2*	1.81	0.91	0.80	1.56	4.25
*LD-CBCT R2 2*	1.80	0.93	0.80	1.42	4.28

Protocols	ICC				
**(b)**					
*HD-CBCT R1 1 vs. HD-CBCT R1 2*	0.904				
*HD-CBCT R2 1 vs. HD-CBCT R2 2*	0.992				
*LD-CBCT R1 1 vs. LD-CBCT R1 2*	0.987				
*LD-CBCT R2 1 vs. LD-CBCT R2 2*	0.991				
*HD-CBCT R1 vs LD-CBCT R1*	0.988				
*HD-CBCT R2 vs LD-CBCT R2*	0.991				
*HD-CBCT R1 vs. HD CBCT R2*	0.992				
*LD-CBCT R1 vs LD-CBCT R2*	0.987				

*Std Dev* standard deviation, *Min* minimum, *Max* maximum, *R1* rater 1, *R2* rater 2, *1* first measurement, *2* s measurement.

HD-CBCT and LD-CBCT identified furcation defects in 41 sites at 19 molars. Eighteen sites with grade III furcation defects were observed at eight teeth (three upper and five lower molars) and 23 sites with grade I and II furcation defects were present at 11 molars (five upper and six lower molars).

Comparison of HD-CBCT with surgical measurement of furcation defects showed a Cohen’s kappa of 0.5975. Comparison of LD-CBCT with surgical measurement of furcation defects also revealed a Cohen’s kappa of 0.5975. All furcation defects identified by HD-CBCT were also identified by LD-CBCT by both raters, giving a Cohen’s kappa of 1.

Linear measurements (f) of the furcation defects showed an interrater agreement (ICC) of 0.992 for HD-CBCT and 0.987 for LD-CBCT. The intrarater agreement (ICC) was 0.994(r1)/0.992(r2) for HD-CBCT and 0.987(r1)/0.991(r2) for LD-CBCT. The intermodality agreement was 0.988(r1)/0.991(r2). These data are presented in Table 1. Paired t-test showed no significant differences between HD-CBCT and LD-CBCT measurements. (Table 2).

**Table 2:** Differences in mean f values.

Protocols tested		p-value
*HD-CBCT R1 1*	*LD-CBCT R1 1*	0.1028
*HD-CBCT R1 2*	*LD-CBCT R1 2*	0.6123
*HD-CBCT R2 1*	*LD-CBCT R2 1*	0.5928
*HD-CBCT R2 2*	*LD-CBCT R2 2*	0.7248
*HD-CBCT R1 1*	*HD-CBCT R2 1*	0.6297
*HD-CBCT R1 2*	*HD-CBCT R2 2*	0.8532
*LD-CBCT R1 1*	*LD-CBCT R2 1*	0.4877
*LD-CBCT R1 2*	*LD-CBCT R2 2*	0.7419
*HD-CBCT R1 1*	*HD-CBCT R1 2*	0.1743
*HD-CBCT R2 1*	*HD-CBCT R2 2*	0.3726
*LD-CBCT R1 1*	*LD-CBCT R1 2*	0.2910
*LD-CBCT R2 1*	*LD-CBCT R2 2*	0.9736

No significant differences in mean f values were observed between HD-CBCT and LD-CBCT according to the paired t-test (p < 0.05).

### Comparison of furcation volume

**Figure 3:**
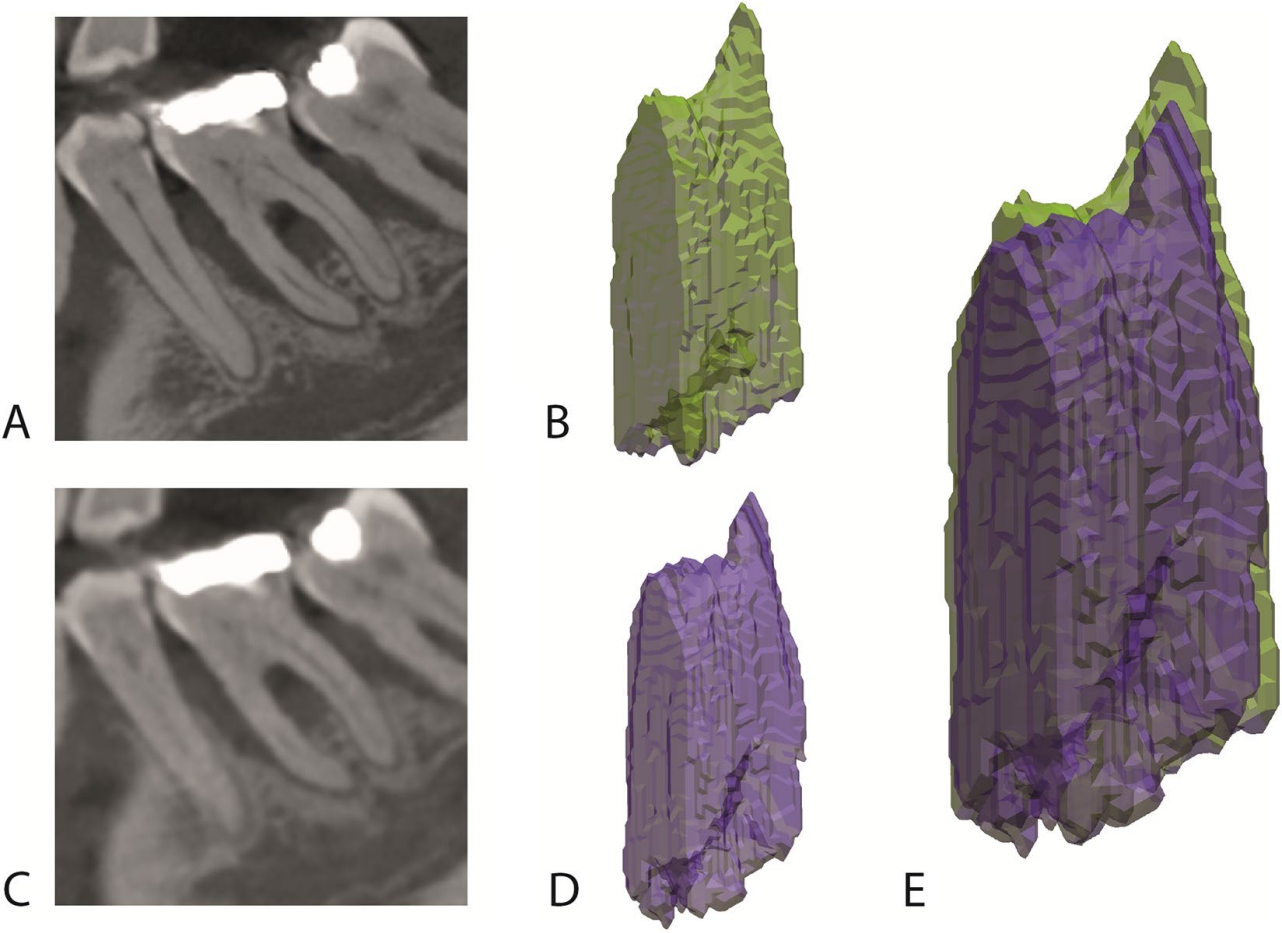
Volume segmentation of the furcation at tooth 36. (**A**) HD-CBCT of tooth 36 in the sagittal plane. (**B**) Corresponding volume model after segmentation and export as STL data into Geomagic Design X. (**C**) LD-CBCT of tooth 36 in the sagittal plane. (**D**) Corresponding volume model after segmentation and export as STL data into Geomagic Design X. (**E**) Overlay of the two STL data sets showing the high volume agreement between HD-CBCT and LD-CBCT.

The furcation volumes measured by HD-CBCT and LD-CBCT showed an intermodality agreement of 0.99 and no significant differences were observed in furcation volumes measured by the two methods (p = 0.09) (Table 3). Figure 3 shows a furcation defect at a mandibular molar constructed from STL data sets after manual segmentation of LD-CBCT and HD-CBCT images.

**Table 3:** Means, medians, ICC-value and p-value of furcation volume measurements.

Protocol	Mean	Std Dev	Min	Median	Max
Volume HD-CBCT	0.0221	0.0516	0.0009	0.0046	0.2591
Volume LD-CBCT	0.0212	0.0498	0.0008	0.0041	0.2511
ICC (volume HD-CBCT vs volume LD-CBCT):	0.99				

Protocols tested	p-value				
Volume HD-CBCT , volume LD-CBCT	0.09				

*Std Dev* standard deviation, *Min* minimum, *Max* maximum.

## Discussion

In this study, we hypothesized that LD-CBCT would detect and visualize furcation defects with the same diagnostic quality as HD-CBCT. Our results confirmed this hypothesis in an ex vivo setting.

We observed high intermodality, interrater, and intrarater agreement, indicating that HD-CBCT and LD-CBCT are both reliable methods for detecting and measuring furcation defects with a sufficient sample size. Compared with intrasurgical measurements of furcation defects, HD-CBCT and LD-CBCT showed moderate Cohen’s kappa values. This is in line with the findings of other studies testing different CBCT protocols for furcation diagnostics^13^. These moderate Cohen’s kappa values may be explained by limitations of the clinical probe used to measure the fine furcation defects. The stiffness and predetermined curvature of the probe may lead to erroneous measurement of a grade II defect instead of a grade III defect in a maxillary molar. This is in line with the findings of Zappa et al., who reported a 27% underestimation of grade III furcation defects measured intrasurgically using the Nabers probe^2^. This underestimation may be due to the shape and diameter of mandibular molars.

The high agreement between HD-CBCT and LD-CBCT in our study shows that (at least when conducted by an experienced examiner) LD-CBCT is just as effective as HD-CBCT at detecting and measuring furcation defects. Earlier studies have already confirmed that HD-CBCT is an accurate method for describing furcation defects^14,15^. Our study goes on to show that LD-CBCT can also accurately describe furcation defects.

This is the first study to investigate detection of furcation defects with a low radiation dose (DAP, 67 mGy cm^2^). The radiation dose we used corresponds to only 7% of that used in the HD-CBCT protocol on the same device^8^. Slightly higher doses were used in earlier studies^13^. For example, digital panoramic views use a DAP of 28 mGy cm^2^, whereas analogue panoramic views use about 88 mGy cm^2^, both of which are higher than the dose used in our LD-CBCT protocol^10^. Another problem with these techniques is that they only represent two dimensions. A full mouth status with digital technology has a DAP of approximately 67 mGy cm^2^, which is within the range used in CBCT, but only represents the structures in two dimensions^10^. This suggests that LD-CBCT could replace the panoramic radiograph in periodontology. Benefits of CBCT to periodontology have been shown, but clinical studies are needed to confirm these benefits^1,16^.

To the best of our knowledge, this is the first study to measure furcation volumes using LD-CBCT. The effect of image quality on the confidence of furcation measurements has already been investigated; the authors concluded that CBCT scan modes significantly affect the confidence of furcation measurements in maxillary molars^13^. In the present study, we measured the volume of furcation defects in an attempt to define the defect margins as precisely as possible. This allowed us to quantify and compare the subjective image quality of HD-CBCT and LD-CBCT. The lower radiation used in LD-CBCT worsens the contrast, but this does not necessarily mean that the contrast is too poor to identify and measure a furcation^17^. We demonstrated good reproducibility in volume measurements between HD-CBCT and LD-CBCT in the present study, indicating that LD-CBCT can produce furcation images of adequate quality compared to HD-CBCT, at least in the hands of an experienced investigator. Image quality that is reduced but sufficient is in line with the ALADA (as low as diagnostically achievable) principle. In addition, segmentation data may be used for automated evaluation programs in the future, allowing evaluation algorithms for LD-CBCT to be developed^18^.

### Limitations

The ex vivo nature leads to a lack of natural motions such as tremor. These motions can lead to motion artifacts, which can significantly reduce image quality and content. Even the human heartbeat can create motion artifacts^17,19^.

The measurements were not fully automated and windowing was allowed, which may have led to voxel interpolation by the software. After software manipulation, the human eye “locates” the bone margin irrespective of the voxel sizes. The different filter settings used could have led to intrarater and interrater differences in the measurements^17^. The surgical measurement of the furcation defects may not have been the optimal reference measurement^2^. However, we used this as a reference measurement to put the LD-CBCT results into clinical context.

Diagnostic interpretation of CBCT results is highly dependent on the experience of the examiners, which means our results may be affected by interrater differences as the examiners in our study had different levels of experience^20^. It has been shown that interrater reliability of marginal bone measurements is lower when the examiners have different levels of experience^21^.

## Conclusions

Overall, our results suggest that LD-CBCT can be used for imaging furcation defects. Within the limitations of the study, our findings show that LD-CBCT is a highly accurate and reliable method for detecting and measuring furcation defects. Further studies should validate these results in a clinical setting before LD-CBCT can complement or replace current procedures for imaging furcation defects such as HD-CBCT or periapical radiographs.

## Supplementary Information

Supplementary Information.

## Data Availability

All data generated or analysed during this study are included in this published article and the supplementary file S1.
